# Gluten-Free Product Recalls and Their Impact on Consumer Trust

**DOI:** 10.3390/nu15194170

**Published:** 2023-09-27

**Authors:** Siyu Liu, Dalia El Khoury, Iris J. Joye

**Affiliations:** 1 Department of Food Science, University of Guelph, 50 Stone Road East, Guelph, ON N1G2W1, Canada; liusiyu9876@gmail.com; 2 Department of Family Relations and Applied Nutrition, University of Guelph, 50 Stone Road East, Guelph, ON N1G2W1, Canada; delkhour@uoguelph.ca

**Keywords:** gluten-free, food recall, celiac disease, non-celiac gluten sensitivity, e-survey, consumer trust

## Abstract

The range of gluten-free food products available to consumers is steadily expanding. In recent years, recalls of food products have highlighted the importance of accurate labeling of food products for the presence of wheat, other gluten-containing cereals, or gluten itself as refined ingredient. The purpose of this study was to gain more insights into recent food recalls related to undeclared gluten/wheat contamination and consumer experiences with these recalls. Recalls of products triggered by gluten contamination are relatively scarce and are not often triggered by a consumer complaint. The impact of these recalls on consumer trust was evaluated through an online survey that was distributed among supporters of Celiac Canada (CCA) and covered (i) strategies to adhere to a gluten-free diet, (ii) experiences with gluten-free recalls and their impact on consumer trust, and (iii) demographic information. Consumer concern regarding gluten-free product recalls is significant, but the concern regarding recalls is not heightened after experiencing a recall. Companies pursuing transparency in the process, identification of the source of contamination, and mitigation strategies going forward are likely to retain consumer trust in their product and brand. Based on the survey results, further efforts focusing on consumer education regarding interpreting nutrient labels, identifying sources of information on product recalls, and understanding procedures to follow upon suspected gluten contamination of a gluten-free product are recommended.

## 1. Introduction

As a highly functional ingredient, wheat gluten gives structure to a variety of bakery and non-bakery food products [1,2]. However, gluten is known to trigger severe immune responses in individuals sensitive to the presence of gluten in food. Celiac disease (CD) e.g., is an autoimmune disorder that triggers both intestinal and extra-intestinal symptoms such as inflammation, villous atrophy, cryptal hyperplasia, and endocrine disorders upon consumption of gluten [3,4,5]. Non-celiac gluten sensitivity (NCGS) has been described as triggering very similar symptoms to CD, both intestinal and extra-intestinal, in patients who improve clinically on a gluten-free diet. However, no specific clinical manifestations or biomarkers have been defined for the diagnosis of NCGS, and a diagnosis is, hence, based on exclusion criteria and the evaluation of symptomatic responses [6]. An estimated 1% of the Canadian population lives with CD while up to 6% of the population may be affected by NCGS [7,8]. A complete and life-long gluten elimination from the diet is to date still the only known effective strategy to ‘treat’ CD and NCGS, tremendously narrowing consumer choices, increasing food costs, and affecting overall consumer well-being by e.g., negatively affecting family activities such as travel and dining out [9]. Recently, about 2% of the Canadian population indicated they follow a gluten-free diet [8].

As the population segment that is currently (or would benefit from) abiding by a strict gluten-free diet is significant and growing [10], gluten replacement in classical cereal and other food products is an ongoing and important challenge for the food production sector. On a global scale, a relatively wide consensus exists that a food may not contain more than 20 ppm of gluten to be labeled as gluten-free [11]. This level is the lowest limit of detection for gluten in foods using scientifically validated analytical methods. As methods evolve and new methods are developed to detect gluten and/or wheat in complex food matrices [12,13,14,15,16], this level may be revised. Gluten-free food was defined in the Codex Alimentarius by the FAO and WHO in the ‘Standard for Foods for Special Dietary Use for persons intolerant to gluten’, first adopted in 1979, revised in 2008, and amended in 1983 and 2015 [17]. Gluten-free food is described by the U.S. Food and Drug Administration (FDA) [18] as food that is either completely gluten-free or that does “not contain (i) an ingredient that is any type of wheat, rye, barley or crossbreeds of these grains, (ii) an ingredient derived from these grains that has not been processed to remove gluten, or (iii) an ingredient derived from these grains that has been processed to remove gluten but results in the food containing more than 20 ppm of gluten.” In Canada, Health Canada [19], based on the available scientific evidence, considers that “gluten-free foods, prepared under good manufacturing practices, which contain levels of gluten not exceeding 20 ppm as a result of cross-contamination, meet the health and safety intent of B.24.018 when a gluten-free claim is made. Based on the enhanced labeling regulations for allergens and gluten sources, any intentionally added gluten sources, even at low levels (e.g., wheat flour as a component in a seasoning mixture which makes up a small proportion of the final food), must be declared either in the list of ingredients or in a “Contains” statement. In these cases, a gluten-free claim would be considered false and misleading. If, however, a manufacturer using a cereal-derived ingredient includes additional processing steps which are demonstrated to be effective in removing gluten, then the food may be represented as gluten-free”.

The production of gluten-free nutritious food products, however, is not a simple, straightforward procedure [20] as is also reflected in gluten-free food recalls that occurred in recent years [21]. A food recall is “the removal of a food from further sale or use, or the correction of its label, at any point in the supply chain as a risk mitigation action”. Some of the triggers that could eventually lead to a food recall are a suspected or confirmed foodborne illness outbreak, a food test result or inspection finding, complaints from consumers/industry/other government departments or associations, a company-initiated recall, a recall in another country, and/or CFIA audit/assessment/evaluation findings [22]. Failure to ensure that all ingredients and all stages of the product manufacturing process are gluten-free is often the basis of gluten-related product contamination and subsequent food recalls. Recalls are a way for food manufacturers to deal with a crisis that poses risks to consumers but simultaneously also affects the financial value and reputation of the company [23].

The objective of this project is to gain more insights into recent food recalls related to undeclared gluten/wheat contamination and consumer experiences with these recalls. The impact of these recalls on consumer trust was evaluated through an online survey that was distributed among supporters of Celiac Canada (CCA). Past studies that link gluten-free product recalls to consumer trust are non-existent. We anticipate, however, that consumer trust will be negatively affected by personal experiences with these recalls. Results from this research will be useful to gain insight in consumer knowledge and choices regarding gluten-free products and recalls, and the course of action that is preferably taken by the affected manufacturer according to consumers to restore their trust in the product and/or manufacturer.

## 2. Materials and Methods

### 2.1. Food Recall Information Collection

Gluten-related product recall data were collected from Health Canada’s dedicated recall webpage after searching the online database with the search term ‘gluten’ and covering the period January 2018—December 2022 [18]. The recalls that were displayed were then checked for duplicate information which was removed prior to frequency analysis.

### 2.2. e-Survey Participant Recruitment

Participants of the survey were recruited through advertisements on social media channels and newsletters of CCA. Prior to completing the survey, participants provided informed consent. An important inclusion criterion for this study was that participants are or had dependents living with CD or NCGS. A total of 1175 valid survey responses were collected. Participants were required to have an adequate literacy of English to complete the questionnaire, had to be over 18 years of age, and were residing in Canada. This project was approved by the Research Ethics Board for compliance with federal guidelines for research involving human participants (REB#21-01-018).

### 2.3. e-Survey Development and Design

The questionnaire was created as an anonymized e-survey on the Qualtrics platform. The questionnaire (Appendix A) included 3 (non-mandatory) parts focusing on (i) strategies to adhere to a gluten-free diet, (ii) experiences with gluten-free recalls, and (iii) demographics. The survey contained a mix of multiple-choice questions and Likert scale questions. In the ‘strategies to adhere to a gluten-free diet’ section, participants were asked how they adhere to a gluten-free diet and how they identify gluten-free food products. In the ‘experiences with gluten-free recalls’ section, questions were asked to probe for the participants’ familiarity with gluten contamination recalls and if they use the provided product information released upon a recall to investigate the food they brought home, where they get their recall information from, and their feelings towards and experiences with gluten contamination-related recalls of gluten-free products. In addition, this survey part also probed for how their trust as a consumer was affected by gluten-related recalls and what manufacturers could do to restore this trust. The questionnaire was tested for content validity and reliability on the target population. Hereto, the questionnaire was sent to a selected group of professionals working for and supporting CCA, and a first survey round was conducted on CCA supporters to assess the survey’s reliability and validity. Input from the group of professionals was utilized to finalize the questionnaire to collect relevant information for future planning and strategy development.

### 2.4. Statistical Analysis

All data analysis was performed using Microsoft Excel, R, and R Studio. Knowledge of and feelings towards gluten contamination-related recalls were estimated overall and within groups formed based on previous recall experiences and compared between these groups using χ2 tests. A *p*-value < 0.01 was considered statistically significant.

## 3. Results

### 3.1. Survey Participants’ Characteristics (Appendix A, Q14–17)

Of the total of 1175 e-survey responses, 36.0% of respondents identified as male, 62.8% identified as female, 0.7% identified as non-binary/third gender, and 0.5% indicated they preferred not to share this information. The majority of the respondents were aged between 25 and 34 years old (Supplementary Table S1) and over two-thirds of the respondents indicated having white/European ethnic backgrounds. More than 5% of the respondents indicated having an indigenous (Aboriginal/First Nations/Métis) ethnic background. Finally, about one-third of the respondents were living in Ontario (31.8%), followed by British Columbia (10.4%), Alberta (9.0%), Manitoba (7.6%), and Quebec (7.5%) (Supplementary Table S2).

### 3.2. Gluten Contamination Recalls in Canada

**Figure 1:**
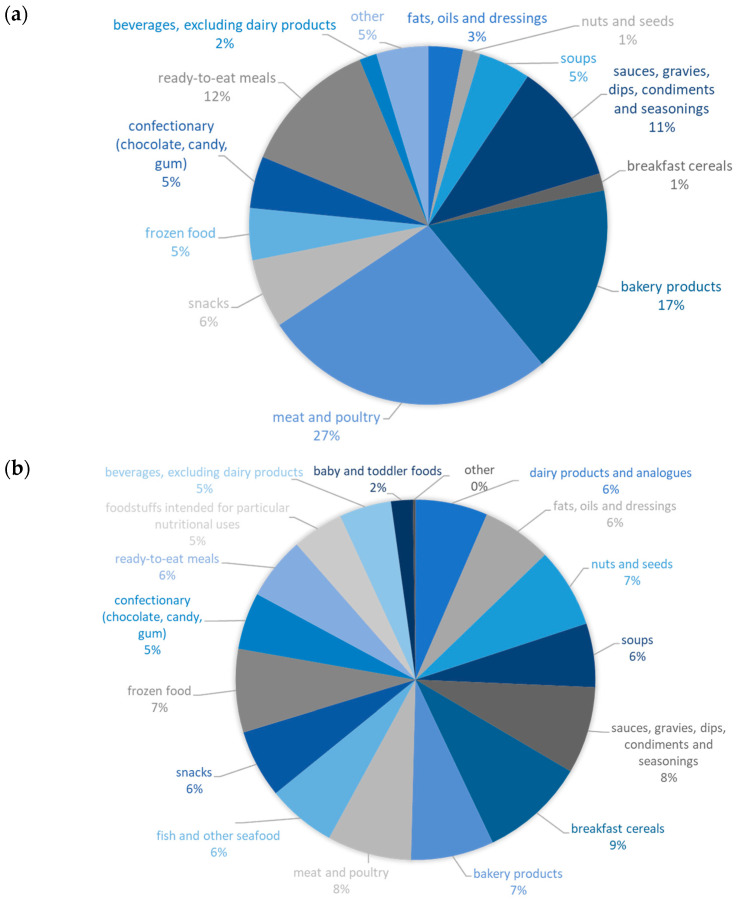
Frequency distribution of product classes implicated in gluten-contamination-related recalls as reported by (**a**) the Government of Canada (reporting period 2018–2022) and (**b**) participants in the gluten-free recall impact survey. The affected products were classified in the following product classes: (i) dairy products and analogs, (ii) fats, oils, and dressings, (iii) nuts and seeds, (iv) soups, (v) sauces, gravies, dips, condiments and seasonings, (vi) breakfast cereals, (vii) bakery products, (viii) meat and poultry, (ix) fish and other seafood, (x) snacks, (xi) frozen food, (xii) confectionary (chocolate, candy, gum), (xiii) ready-to-eat meals, (xiv) foodstuffs intended for particular nutritional uses (e.g., protein shakes, …), (xv) beverages, excluding dairy products, (xvi) baby and toddler foods, (xvii) other.

Gluten is omnipresent in our food chain as it is not only used in the bakery sector but is often also utilized in meat, snacks, or other products as a binder and structure-giving ingredient [15,24]. It is, hence, of utmost importance to declare its presence on the product label. Not only gluten from wheat pose a problem, but also the ethanol-soluble storage proteins of barley, rye, and spelt are known to trigger adverse reactions in people living with CD [25]. As such, failure to declare the presence of wheat, spelt, barley, rye, or gluten itself on a product ingredient list results in food product recalls. Sixty-four recalls due to undeclared gluten, wheat, and barley have been reported on the website of the Government of Canada [21] for the 2018–2022 period. Based on the information provided in the recall documents, these recalls were either triggered by the company (*n* = 9), after a consumer complaint (*n* = 10), or upon inspection by CFIA (*n* = 8). The majority of the recall documents, however, did not contain this information. Most of the recalls during this period were triggered for meat and poultry products (27%, *n* = 17), bakery products (17%, *n* = 11), sauces, gravies, dips, condiments and seasonings (11%, *n* = 7), and ready-to-eat meals (12%, *n* = 8). Products that did not fall into any of the other categories (and are reported as ‘other’ in Figure 1a) were vegetarian meat alternatives (*n* = 3) and potato products (*n* = 1). Not all of the recalled products were advertised and sold as being gluten-free, but the ingredient labels suggested that there was no wheat, barley, and/or gluten present in the product and the labeling could, hence, be seen as misleading. A recent study by Franco-Arellano and coworkers [26] found that about 15% of the prepackaged foods and beverages in the Canadian food supply chain carry gluten-free claims and that these were usually classified as processed or ultra-processed foods. The product categories displaying the largest proportion of gluten-free claims according to this same study are snacks, meat and poultry products, and nuts and seeds, i.e., products that would generally use gluten as a ‘hidden’ ingredient to increase product quality. In that regard, it is not surprising that meat and poultry products are also on top of the gluten contamination-related recall list, often ‘due to undeclared wheat, gluten and/or barley’.

About 80 and 40% of the respondents indicated their gluten-free meals were prepared at home by themselves or someone else, respectively, while about 36% also reported buying gluten-free meals from restaurants (including school cafeterias and unit restaurants) (Appendix A, Q1). About 70% of the respondents indicated to have been personally affected by gluten contamination-related recalls and these respondents were asked which category of product was involved in the recall (Appendix A, Q6–7). The product categories reported by the respondents in the survey were more diverse and did not overlap well with the recall information from the Government of Canada (Figure 1b). However, the classification of food products is not straightforward as some products may fit more than one category and the respondents may have experienced gluten recalls prior to the reported period (2018–2022) or outside Canada.

In addition, as outlined earlier, a substantial number of recalls were initiated either by the manufacturer itself or CFIA and did not get triggered by a consumer complaint. As reported by the respondents, affected product classes included breakfast cereals (9%, *n* = 281), sauces, gravies, dips, condiments and seasonings (8%, *n* = 232), meat and poultry products (8%, *n* = 225), bakery products (7%, *n* = 222), frozen food products (7%, *n* = 222), and nuts and seeds (7%, *n* = 213). Besides the products that traditionally have cereals included in the recipe, meat products, sauces, gravies, dips, condiments, and seasonings were also identified as food products affected by recalls by both the survey respondents and the recall information. It, is hence, important that people living with CD and gluten intolerance remain vigilant when purchasing a wide range of food products. This constant vigilance, combined with the higher cost of gluten-free products, puts a large strain on consumers following a gluten-free diet [6]. Food producers have a large responsibility to ensure that gluten-free products are truly gluten-free and that gluten, wheat, barley, rye, and/or spelt present in food products are clearly declared. Survey respondents indicated to rely on ingredient lists (76%), nutrient-related claims (e.g., ‘certified gluten-free’ label on the food package, 72%) and own expertise (29%) to identify gluten-free products (Table 1) (Appendix A, Q5). 4% of the respondents indicated that they do not check for gluten-free information on the products they eat (Table 1). As an estimated 6% of the Canadian population lives with CD or gluten intolerance, and the population segment following a gluten-free diet is still growing [10], it is important to probe for consumer awareness, experience, and knowledge concerning gluten contamination and associated recalls.

### 3.3. Feelings and Experiences Regarding Gluten-Free Recalls

About 97% of the respondents indicated they had heard about gluten contamination, while close to 70% reported to have been personally affected by a gluten contamination-related recall (Table 2) (Appendix A, Q2&6). Almost 79% of the respondents indicated to be at least somewhat concerned regarding recalls of gluten-free products due to gluten contamination (Table 2) (Appendix A, Q4). Channels through which information on recalls of gluten-free products due to gluten contamination are obtained are social media (Facebook, Instagram, etc., 73%), news (TV, radio, newspaper, etc., 50%), government publications (36%) and family and friends (35%) (Table 1) (Appendix A, Q3). About 6% of the respondents indicated they did not receive this information. Releases from both CCA and CFIA were often mentioned as good sources of information. The above indicates that the respondents, and likely the wider population living with CD or gluten intolerances, are generally well-informed and know how to access information crucial to their commitment to a gluten-free diet. When a food is recalled, more information is usually provided in terms of lot numbers and date of production of the affected product. 62% of the respondents indicated that they checked this information prior to eliminating the product (Table 1) (Appendix A, Q8).

The survey results indicated that there is no dependence (*p* > 0.01) between having been personally affected by a gluten-contaminated recall of gluten-free products and concern feelings on this topic (Table 3) (Appendix A, Q4 and 6). Trust in a food manufacturing company that has gone through a recall and the likelihood of purchasing a product that has been implicated in a recall before, however, did show a dependence on previous personal experiences with gluten contamination-related recalls (*p* < 0.01, Table 3) (Appendix A, Q6&9–11). Quite surprisingly, however, it seems that the likelihood of trusting a company implicated in a food recall was actually lower for people who did not personally experience a gluten contamination-related recall, and respondents who did experience a recall before were more trusting of affected manufacturing companies. The same unexpected trend in responses was observed for the question probing for the likelihood of purchasing a product that was previously recalled due to gluten contamination (Table 3).

### 3.4. Recommendations for Industry and Policy Makers

When a recall is required for one of the products based on gluten contamination, the majority of the respondents in this study (64%) indicated that the best strategy to restore trust after a gluten-free recall of a gluten-free product would be full disclosure on the source of the problem and possible mitigation strategies taken (Table 4) (Appendix A, Q12). In addition, alternative communication strategies such as an apology (58%) or the offering of compensation (e.g., monetary compensation or coupon) (53%) were also seen as effective strategies by the majority of the respondents (Table 4). It is of note that 11% of the respondents indicated that they would retain trust in the company regardless of the recall, while about 4% indicated that there would be no strategy effective to restore their consumer trust (Table 4).

Finally, about 20% of the respondents indicate that they do not know what to do if they suspect a product labeled as not containing gluten to be contaminated by gluten (Table 4) (Appendix A, Q13).

## 4. Discussion

Although participants were recruited through CCA, the surveyed respondent population was found not to be a perfect representative cross-section of the Canadian population segment affected by CD and NCGS, as the participants were largely of white/European ethnicity (~70%) and living in Ontario (32%). This may have affected the responses that were obtained, and care must be taken when extrapolating the results to the entire population living with CD and NCGS in Canada. Nevertheless, it has been reported that the diagnosis of CD in females is higher and women constitute typically 60–70% of the individuals diagnosed with CD [2], which is in line with 70% of the survey respondents identifying as female. However, it is unclear if the higher diagnosis numbers in females are largely driven by an actual higher prevalence of CD in females or if this is related to the fact that females more frequently use healthcare services than males or because women show more gastrointestinal symptoms and clinical signs associated to CD than males do [27,28,29]. As for ethnic background, differences in CD prevalence were suggested. Mardini and colleagues [30] e.g., found that the prevalence of CD in non-Hispanic white people was higher than what was found for other races. However, more population-based prevalence studies need to be carried out to fully shed light on the CD and NCGS prevalence [28]. In this regard, our survey respondent population seemed to have reached the most affected subpopulations, i.e., females and people of white/European ethnicity, according to current diagnosis/prevalence numbers.

Since gluten is not only present in food through the use of certain cereal flours (i.e., wheat, rye, spelt, and/or barley) but is also used in an isolated form as e.g., a texturizing or water-holding ‘hidden’ ingredient, it is often difficult to identify gluten and/or assess the risk of gluten contamination in a food product [31,32]. Adhering to a gluten-free diet puts strain on consumers while dining out, as in a social context adhering to such a diet may be perceived to result in reputational damage [9,33]. In this context, Silvester et al. [34] stated that having to adhere to a gluten-free diet leads to mild impairment in social leisure activities, a shift to eating more meals at home, and in rare instances negative emotions such as anxiety, isolation, and frustration. In addition, a more recent study by Lebovits and colleagues [35] indicated that CD also impacts dating behavior, decreases the quality of life, and leads to greater social anxiety. A study in 2006 showed that almost 79% of the respondents living with CD avoid restaurants [36]. In the presented study, more than one-third of the respondents indicate they obtain their food from outside-home settings such as a restaurant or school cafeteria. Since 2006, more education has led to an increased awareness among people not following a gluten-free diet of the importance of following this strict diet for people living with CD and NCGS. In addition, in response to more people adhering to a perceived healthier gluten-free lifestyle, the demand for but also the offering of gluten-free food options has increased significantly over the past couple of years [10,37]. However, it is rare to see restaurants that only offer gluten-free meals, as most restaurants do offer separate gluten-free menus or simply indicate whether the dish is gluten-free or not on their menu.

As outlined earlier, in Canada, gluten must be declared on the food label of a product when being used as an ingredient [38]. Besides identifying gluten-free food products by ingredient labels (76%), 72% of the respondents also check nutrient-related claims such as gluten-free labels on the food package, underlining the importance of the accuracy of the information on the packaging. Previous research already indicated that consumers living with CD may be struggling to choose gluten-free foods based on product labeling [18]. In conclusion to their research, these authors recommended a more explicit identification of gluten-free products. Studies by Silvester and colleagues [39] and Dowhaniuk and coworkers [40], revealed that gluten ingestion frequently occurs despite efforts of consumers to follow a strict gluten-free diet. It seems that complete elimination of gluten is often impossible to maintain and in reality, most patients with CD follow a low-gluten diet [39]. The gluten limit, set to <20 ppm by governmental organizations, illustrates an explicit allowance for gluten in a gluten-free diet, although this level originates from a limit of detection of gluten in complex matrices as mentioned earlier. Koerner et al. [3] conducted a gluten quantification study on gluten-free ingredients and confirmed that these ingredients labeled gluten-free are also the safest food products for individuals with CD and NCGS to consume. Naturally gluten-free products with no ‘gluten-free’ label are likely to have a higher risk of gluten contamination [3,41]. Similarly, research conducted in the U.S. also reported that consumers can rely on gluten-free labels when identifying gluten-free foods, where 95% of gluten-free labels are trustworthy [42]. In addition, the occurrence of gluten contamination of foods has declined over time [41]. However, the risk of cross-contamination with gluten is still possible, posing potential risks to those who are gluten intolerant [43]. In this context, the Gluten-Free Certification Program (GFCP) endorsed by CCA seems to be well-known and trusted to provide protection to consumers and guidance on differentiating gluten-free certified products [44].

Only 2.7% of the respondents indicated they had not heard of ‘gluten contamination’ before. About two-thirds of the respondents had been personally affected by previous gluten contamination-related recalls of gluten-free products. Quite surprisingly, consumer trust in affected companies and/or purchase behavior regarding affected products was not negatively impacted by the recall experiences. Quite conversely, the group that was personally affected by a gluten-free product recall overall seemed to be more trusting of the companies and indicated a higher likelihood of purchasing the affected product again. When probing for strategies to rebuild trust, only a small number of respondents (4.2%) indicated that their trust would never be restored after a recall. Some participants indicated that the history of previous recalls by the same company and the brand trust existing prior to the recall play a big role in retaining trust in the affected manufacturer. Yakut & Bayraktaroglu [45] reported that firm reputation is indeed an important factor, where a company with better reputation may better protect themselves from severe impacts of a food recall crisis and that food companies with higher reputation will be more efficient than companies with lower reputation in crisis management. As mentioned earlier, some of the gluten contamination-related recalls were initiated by the companies themselves, so-called voluntary product recalls. Of the above 64 identified recalls between 2018 and 2022, at least 9 were voluntary recalls. Bortoli and Freundt [23] found that a voluntary recall generates a positive impact on consumer’s trust perceptions of an organization. As such, in a crisis triggered by product failures pursuing a voluntary recall despite the associated risks and costs, is still recommended. In general, more transparency on food recalls could make the food value chain even more safe and trusted by consumers. For food manufacturers that have gone through a recall, it is clear from the survey results that they need to take responsibility for any mistakes they made leading to the recall, if any. Disclosing the problem, increased transparency on mitigation strategies, and a more open and straightforward communication targeted towards consumers (64%) and an apology (58%) were the top strategies put forward through the survey that could help restore consumer trust. Offering compensation to consumers (53%) was only the third-most reported trust-rebuilding strategy by the survey respondents.

About 73% of the survey respondents indicated they receive information regarding gluten contamination recalls of gluten-free products through social media. The connectedness of people through social media creates opportunities for the quick sharing of news and the promotion of social learning [46]. However, the participant recruitment for this survey was also carried out through social media, which may have biased the responses to this question. Of note is also that 67 respondents indicated they do not receive information on gluten-free recalls (despite virtually all participants indicating that they are at least somewhat concerned about gluten-free recalls), which indicates that the method to obtain such information can still be made more accessible and consumers can still be better educated on how this information can be obtained and should be used/interpreted.

Lastly, more than four-fifths of the respondents (80.3%) indicated that they know what the to-follow procedure is when they suspect a gluten-free product to be contaminated with gluten. Increased exposure to procedural information via, e.g., social media and government-run resources should further increase procedural insights. Non-profit organizations such as CCA, where a significant share of the respondents received their information from, could also provide guidance on this topic.

## 5. Conclusions

In conclusion, recalls of products triggered by gluten contamination are relatively scarce and are often not triggered by a consumer complaint. Consumer concern regarding these recalls is significant, but the concern regarding recalls is not heightened after experiencing a recall. Companies pursuing transparency in the process, identification of the source of contamination, and mitigation strategies going forward are likely to retain consumer trust in their product and brand. More effort could be dedicated to consumer education regarding reading nutrient labels, sources of information on product recalls, and procedures to follow upon suspected gluten contamination of a gluten-free product.

## Figures and Tables

**Table 1:** Frequency analysis of survey replies regarding (i) strategies to identify gluten-free food products, (ii) platforms through which recall information is obtained, (iii) investigation of the lot numbers/production date of affected products prior to the elimination of the product (n_total_ = 1175).

Question	Answer	*n*	Percentage
What do you use to identify gluten-free food products? Check all that apply. (Appendix A, Q5)	Nutrient-related claim (e.g., ‘certified gluten-free label’) on the food package	842	71.7
	Ingredient list	892	75.9
	Own expertise	336	28.6
	I do not check at all	47	4.0
	Other	16	1.4
	No answer	2	0.2
Through which platform do you receive information about recalls of gluten-free products due to gluten contamination? Check all that apply. (Appendix A, Q3)	News (TV, radio, newspaper, etc.)	588	50.0
	Social media (Facebook, Instagram, etc.)	854	72.7
	Website	78	6.6
	Family and Friends	410	34.9
	Government publications	423	36.0
	Non-government publications	13	1.1
	I do not receive information	67	5.7
	Other	21	1.8
	No answer	33	2.8
When a food product is recalled, often more information is provided in terms of lot numbers and date of production of the affected product. Do you investigate the above mentioned specifics of the recall prior to eliminating the product? (Appendix A, Q8)	Yes	723	61.6
	No	76	6.5
	No answer	375	31.9

**Table 2:** Awareness, experiences, and knowledge about gluten-free recalls (*n* (%), n_total_ = 1175).

Question	Answer: Yes	Answer: No	No Answer
Have you heard of gluten contamination? (Appendix A, Q2)	1142 (97.2%)	32 (2.7%)	1 (0.1%)
Have you been personally affected by gluten-contamination-related recalls in gluten-free products? (Appendix A, Q6)	810 (68.9%)	362 (30.8%)	3 (0.3%)
Would you know what to do if you suspect a product labelled as not containing gluten to be contaminated with gluten? (Appendix A, Q13)	944 (80.3%)	229 (19.5%)	2 (0.2%)

**Table 3:** Frequency analysis of survey replies (*n*, %) regarding personal experience with gluten-free product recalls and (A) feelings of concern regarding gluten-free product recalls, (B) trust in affected companies, and (C) willingness to purchase an affected product again. Only those responses in the survey that addressed both questions are listed in this table. χ2 scores and *p*-values report on the dependence between personal experiences with recalls, on the one hand, and feelings, trust, and likelihood to purchase, on the other hand.

Have You Been Personally Affected by Gluten Contamination-Related Recalls in Gluten-Free Products? (Appendix A, Q6)				
		No	Yes	Total
A. How do you feel about recalls of gluten-free products due to gluten contamination? (Appendix A, Q4) χ2 = 12.638, *p* = 0.01319	Extremely concerned	107 (30.0%)	209 (30.0%)	316 (28.6%)
	Moderately concerned	79 (22.2%)	193 (25.8%)	272 (24.7%)
	Somewhat concerned	94 (26.4%)	187 (25.0%)	281 (25.5%)
	Slightly concerned	68 (19.1%)	156 (20.9%)	224 (20.3%)
	Not at all concerned	8 (2.2%)	2 (0.3%)	10 (0.9%)
	GRAND TOTAL	356	747	n_total_ = 1103
B. How likely are you to trust a food manufacturing company if you know it has gone through a recall due to gluten contamination of a gluten-free product before? (Appendix A, Q11) χ2 = 66.432, *p* = 1.284 × 10^−13^	Extremely likely	4 (1.1%)	45 (5.6%)	49 (4.2%)
	Somewhat likely	44 (12.2%)	240 (29.8%)	284 (24.3%)
	Neither likely or unlikely	102 (28.2%)	209 (25.9%)	311 (26.6%)
	Somewhat unlikely	160 (44.2%)	240 (29.8%)	400 (34.2%)
	Extremely unlikely	52 (14.4%)	72 (8.9%)	124 (10.6%)
	GRAND TOTAL	362	806	n_total_ = 1168
C. How likely are you to purchase a gluten-free product if you know it had been recalled due to gluten contamination before? (Appendix A, Q9/10) χ2 = 71.156, *p* = 1.294 × 10^−14^	Extremely likely	20 (5.5%)	71 (8.8%)	91 (7.8%)
	Somewhat likely	50 (13.8%)	279 (34.4%)	329 (28.1%)
	Neither likely or unlikely	102 (28.2%)	184 (22.7%)	286 (24.4%)
	Somewhat unlikely	119 (32.9%)	198 (24.4%)	317 (27.1%)
	Extremely unlikely	71 (19.6%)	77 (9.5%)	148 (12.6%)
	GRAND TOTAL	362	809	n_total_ = 1171

n_total_: total number of respondents that submitted a response to both questions.

**Table 4:** Frequency analysis of survey replies regarding (i) preferred strategies to restore consumer trust after a gluten-related recall, and (ii) procedure to follow when a product labeled not to contain gluten is suspected to be contaminated with gluten (n_total_ = 1175).

Question	Answer	*n*	Percentage
What strategies of the below do you feel would be more effective in restoring your consumer trust after a gluten-related recall of a gluten-free product? Check all that apply. (Appendix A, Q12)	Communication strategies (e.g., an apology)	686	58.4
	Compensation (e.g., a monetary compensation or coupon)	627	53.4
	Disclosure of the source of the problem and possible mitigation strategies	750	63.9
	I trust the company regardless of the gluten contamination recall	128	10.9
	No strategy would be effective in restoring my consumer trust	49	4.2
	Other	13	1.1
	No answer	1	0.1
Would you know what to do if you suspect a product labelled as not containing gluten to be contaminated with gluten? (Appendix A, Q13)	Yes	944	80.3
	No	229	19.5
	No answer	2	0.2

## Data Availability

The data presented in this study are available on request from the corresponding author. The data are not publicly available due to ethical restrictions.

## Supplementary Materials

The following supporting information can be downloaded at: https://www.mdpi.com/article/10.3390/nu15194170/s1, Table S1: Age distribution of gluten-free recall survey respondents; Table S2: Province/territory distribution of gluten-free recall survey respondents.

## Appendix A

**Q1. How do you adhere to the gluten-free diet? Check all that applies.**

- I cook my own gluten-free meals
- Someone at home prepares gluten-free meals for me
- I buy gluten-free meals from restaurants (including school cafeterias and unit restaurants)
- Other

Please provide details

**Q2. Have you heard of gluten contamination?**

- Yes (respondents are directed to question 3)
- No (definition is shown (Gluten is a protein naturally found in some grains such as wheat, barley, and rye. Gluten contamination refers to the presence of gluten in gluten-free food products), and respondents are directed to question 4)

**Q3. Through which platform do you receive information about recalls of gluten-free products due to gluten contamination? Check all that apply.**

- News (TV, radio, newspaper etc.)
- Social Media (Facebook, Instagram etc.)
- Website

Please specify the website

- Family and friends
- Government publications
- Non-government publications

Please specify the publication

- I do not receive information
- Other

Please provide details

**Q4. How do you feel about recalls of gluten-free products due to gluten contamination?**

- Not at all concerned
- Slightly concerned
- Somewhat concerned
- Moderately concerned
- Extremely concerned

**Q5. What do you use to identify gluten-free food products? Check all that apply.**

Nutrient-related claim (e.g., ‘certified gluten-free label’) on the food package

- Ingredient list
- Own expertise
- I do not check at all
- Other

Please provide details

**Q6. Have you been personally affected by gluten contamination-related recalls in gluten-free products?**

- Yes (respondents are directed to questions 7 to 9)
- No (respondents are directed to question 10)

**Q7. What product categories did the recalled gluten-free products belong to?**

- Dairy products and analogues
- Fats, oils and dressings
- Nuts and Seeds
- Soups
- Sauces, gravies, dips, condiments and seasonings
- Breakfast cereals
- Bakery products
- Meat and poultry
- Fish and other seafood
- Snacks
- Frozen food
- Confectionary (chocolate, candy, gum)
- Ready-to-eat meals
- Foodstuffs intended for particular nutritional uses (e.g., protein shakes, …)
- Beverages, excluding dairy products
- Baby and toddler foods
- Other

Please provide details

**Q8. When a food product is recalled, often more information is provided in terms of lot numbers and date of production of the affected product. Do you investigate about the above mentioned specifics of the recall prior to eliminating the product?**

- Yes
- No

**Q9. How likely will you purchase a gluten-free product again if you know it has been recalled due to gluten contamination before?**

- Extremely unlikely
- Somewhat unlikely
- Neither likely nor unlikely
- Somewhat likely
- Extremely likely

**Q10. How likely will you purchase a gluten-free product if you know it had been recalled due to gluten contamination before?**

- Extremely unlikely
- Somewhat unlikely
- Neither likely nor unlikely
- Somewhat likely
- Extremely likely

**Q11. How likely are you going to trust a food manufacturing company if you know it has gone through a recall due to gluten contamination of a gluten-free product before?**

- Extremely unlikely
- Somewhat unlikely
- Neither likely nor unlikely
- Somewhat likely
- Extremely likely

**Q12. What strategies of the below do you feel would be more effective in restoring your consumer trust after a gluten-related recall of a gluten-free product? Check all that apply.**

- Communication strategies (e.g., an apology)
- Compensation (e.g., a monetary compensation or coupon)
- Disclosure on the source of the problem and possible mitigation strategies
- I trust the company regardless of the gluten contamination recall
- No strategy would be effective in restoring my consumer trust
- Other

Please provide details

**Q13. Would you know what to do if you suspect a product labelled as not containing gluten to be contaminated with gluten?**

- Yes
- No

**Q14. What is your gender?**

- Man
- Woman
- Non-binary/third gender
- Prefer not to say
- Prefer to self-describe

**Q15. What is your age group?**

- 18–24
- 25–34
- 35–44
- 45–54
- 55–64
- 65–74
- 75+
- Prefer not to answer

**Q16. Which of the following BEST describes your ethnic background? Check all that apply.**

- Indigenous (Aboriginal/First Nations/Métis)
- White/European
- Black/African/Caribbean
- Southeast Asian (e.g., Chinese, Japanese, Korean, Vietnamese, Cambodian, Filipino, etc.)
- Arab (Saudi Arabian, Palestinian, Iraqi, etc.)
- South Asian (East Indian, Sri Lankan, etc.)
- Latin American (Costa Rican, Guatemalan, Brazilian, Columbian, etc.)
- West Asian (Iranian, Afghani, etc.)
- Other (please specify)
- Prefer not to answer

**Q17. Which province or territory do you live in?**

- Alberta
- British Columbia
- Manitoba
- New Brunswick
- Newfoundland and Labrador
- Northwest Territories
- Nova Scotia
- Nunavut
- Ontario
- Prince Edward Island
- Quebec
- Saskatchewan
- Yukon
- Prefer not to answer
