# Supplementary material for: Gluten-Free Product Recalls and Their Impact on Consumer Trust
Source: Nutrients. 2023 Sep 27;15(19):4170. doi: 10.3390/nu15194170 (PMC10574315; doi:10.3390/nu15194170)
Supplement: Supplementary file 1 [file nutrients-15-04170-s001.zip › nutrients-2616826-supplementary.pdf]

Supplementary material

Table S1: Age distribution of gluten-free recall survey respondents (n=1175).

Age range	Number	Percentage (%)
18-24	147	12.5
25-34	486	41.4
35-44	307	26.2
45-54	164	14.0
55-64	48	4.1
65-74	14	1.2
75+	4	0.3
Prefer not to answer/blank	5	0.3

Table S2: province/territory distribution of gluten-free recall survey respondents (n=1175).

Province	Number	Percentage (%)
Alberta	106	9.0
British Columbia	122	10.4
Manitoba	89	7.6
New Brunswick	65	5.5
Newfoundland and Labrador	61	5.2
Northwest Territories	52	4.4
Nova Scotia	54	4.6
Nunavut	35	3.0
Ontario	373	31.8
Prince Edward Island	31	2.6
Quebec	88	7.5
Saskatchewan	32	2.7
Yukon	27	2.3
Prefer not to answer/blank	40	3.3
